# A red nodule in the umbilicus of an older man

**DOI:** 10.1093/skinhd/vzaf018

**Published:** 2025-04-29

**Authors:** Sarah Winter, Sally Ashton, Daniel Housa, Avad Mughal

**Affiliations:** Department of Dermatology, Neath Port Talbot Hospital, Port Talbot, UK; School of Medicine, Cardiff University, Cardiff, UK; Department of Dermatology, Neath Port Talbot Hospital, Port Talbot, UK; Department of Pathology, Morriston Hospital, Swansea, UK; Department of Dermatology, Neath Port Talbot Hospital, Port Talbot, UK

## Abstract

This case report describes an interesting example of a syringocystadenoma papilliferum lesion presenting in a 72-year-old man. He presented with a 10-month history of a red nodule in his umbilicus with unexplained weight loss and reduced appetite. The patient had a background of chronic obstructive pulmonary disease, treated prostate cancer and pemphigus vulgaris that had previously been treated with azathioprine. The nodule would occasionally bleed with trauma, but there were otherwise no associated symptoms. Physical examination revealed a 12 × 12 mm firm red nodule within the umbilicus with some creamy exudate overlying it. Given the clinical presentation, differentials at the time included Sister Mary Joseph nodule, amelanotic melanoma and pyogenic granuloma. A shave biopsy was arranged to help diagnose the nodule and further investigations including computed tomography (CT) and colonoscopy were undertaken. CT and colonoscopy did not indicate any sinister pathology. Histopathological findings demonstrated mildly cystic invaginations arising from a papillomatous epidermis that were lined by rows of cuboidal-to-columnar epithelial cells, with oval nuclei and a pale eosinophilic cytoplasm with squamous metaplasia. The stroma contained a dense mononuclear infiltrate, which was comprised predominantly of plasma cells and lymphocytes. The histopathological findings were of syringocystadenoma papilliferum. This report discusses the clinical and histopathologicial presentation of syringocystadenoma papilliferum and the investigations and management to consider with this diagnosis. We also discuss the various differentials that should be considered for a red nodule in the umbilicus.

## Teaser

This case report is the first in literature to document a syringocystadenoma papilliferum occurring in an umbilicus. This report discusses the various differentials that should be considered for a red nodule in the umbilicus. We also discuss the clinical and histopathologicial presentation of syringocystadenoma papilliferum, and the investigations and management to consider with this diagnosis.

**What is already known about this topic?**

- Syringocystadenoma papilliferum is a rare tumour that usually affects the head and neck.
- It often develops congenitally or during puberty.
- Complete surgical excision is recommended in case reports as management.

**What does this study add?**

- We present a case of syringocystadenoma papilliferum in an unusual anatomical area.
- We highlight this rare tumour for clinical and histological identification.
- Successful surgical management adds to literature to strengthen evidence for complete excision.

## Case report

### Clinical findings

**Figure 1:**
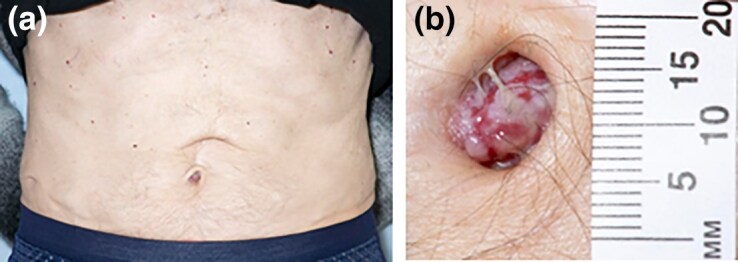
(a) Red nodule in patient’s umbilicus. Scar also visible above the umbilicus. (b) Close-up view of the nodule.

A 72-year-old man presented with a 10-month history of a red nodule in his umbilicus with weight loss and reduced appetite. The patient had a background of pemphigus vulgaris (previously treated with azathioprine but currently on a slow weaning dose of prednisolone), chronic obstructive pulmonary disease and treated prostate cancer. The nodule would occasionally bleed with trauma, but there were otherwise no associated symptoms. Physical examination revealed a 12 × 12 mm firm red nodule within the umbilicus with some creamy exudate overlying it (Figure 1). A surgical scar was noted above the umbilicus. A shave biopsy was taken and sent for histopathological examination. Computed tomography (CT) and colonoscopy did not indicate any sinister pathology.

### Histopathological findings

**Figure 2:**
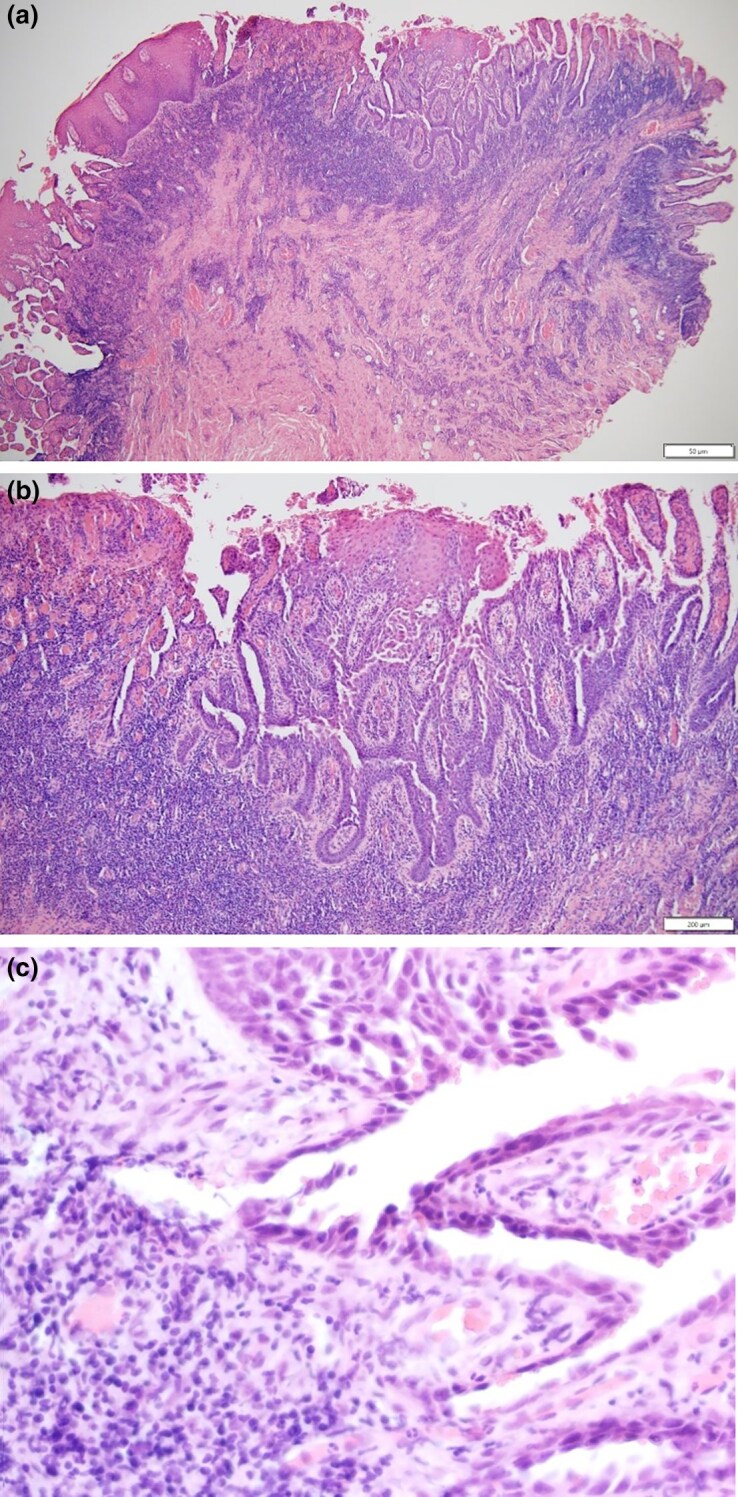
(a) Haematoxylin and eosin original magnification × 40: general representative view; (b) haematoxylin and eosin original magnification × 100: invaginations of infundibular epithelium from a papillomatous epidermis extending into the dermis; (c) haematoxylin and eosin original magnification × 200: closer view of invaginations and stroma containing dense mononuclear infiltrate comprised predominantly of plasma cells and lymphocytes.

Mildly cystic invaginations arise from a papillomatous epidermis. These invaginations demonstrate papillae lined by rows of cuboidal-to-columnar epithelial cells, with oval nuclei and a pale eosinophilic cytoplasm with squamous metaplasia (Figure 2). Rare dermal ductal component was noted. The stroma contains a dense mononuclear infiltrate, which is comprised predominantly of plasma cells, lymphocytes and rare scattered neutrophils.

## Discussion

Syringocystadenoma papilliferum (also known as papillary syringadenoma) is a rare, benign hamartomatous adnexal tumour. This tumour is commonly seen in the head and neck area.^1^ Several unusual anatomical sites of involvement have been reported, including arms, eyelids and perianal regions.^2^ Our case is the first known to arise in the umbilicus. It is often found congenitally or seen to develop during puberty.^2^ Clinically, syringocystadenoma papilliferum present as slow-growing, predominantly asymptomatic, hairless lesions. They are rarely pigmented and may be skin-coloured or erythematous. They may resemble other skin lesions such as pyogenic granuloma, squamous cell carcinoma or amelanotic melanoma. In comparison with the other differentials, syringocystadenoma tends to be slow growing. In our patient, given the location and clinical history, Sister Mary Joseph nodule was also a differential. Three clinical types have been described: plaque, linear and solitary nodular type. Due to its nonspecific appearance and rarity, diagnosis requires histopathological examination.

The tumour originates from the apocrine or eccrine sweat glands; histopathological features include cystic invaginations of infundibular epithelium that disrupt the dermis with a double layer of columnar and cuboidal cells. Proliferating and variable glandular tissue and infiltrative plasma cells are commonly seen alongside papillary epidermal hyperplasia with hyperkeratosis and hypergranulosis.^1,3,4^ Differential diagnosis includes cutaneous adnexal tumours with a glandular/ductal component, including hidradenoma papilliferum, tubular adenoma, hidradenoma, naevus sebaceus and apocrine mixed tumour. When squamous metaplasia and/or epidermal/infundibular hyperplasia are prominent, the lesion can be misdiagnosed as keratoacanthoma or even possibly verrucous squamous cell carcinoma.

Syringocystadenoma papilliferums are predominantly benign; however, cases of malignant transformation have been reported. Therefore, the most favoured management of syringocystadenoma papilliferum is complete surgical excision. Mohs surgery and carbon dioxide laser have also been used successfully.^5,6^ Postdiagnosis, our patient underwent complete surgical excision to complete ­treatment.

## Data Availability

No new data were generated or analysed in support of this research.
